# Self-consciousness concept and assessment in self-report measures

**DOI:** 10.3389/fpsyg.2015.00930

**Published:** 2015-07-03

**Authors:** Amanda DaSilveira, Mariane L. DeSouza, William B. Gomes

**Affiliations:** ^1^Laboratory of Experimental Phenomenology and Cognition, Institute of Psychology, Universidade Federal do Rio Grande do SulPorto Alegre, Brazil; ^2^Graduate Program in Psychology, Universidade Federal do Espírito SantoVitória, Brazil

**Keywords:** self-consciousness, self-awareness, self-reflection, mindfulness, self-absorption, self-rumination, self-report measures

## Abstract

This study examines how self-consciousness is defined and assessed using self-report questionnaires (Self-Consciousness Scale (SCS), Self-Reflection and Insight Scale, Self-Absorption Scale, Rumination-Reflection Questionnaire, and Philadelphia Mindfulness Scale). Authors of self-report measures suggest that self-consciousness can be distinguished by its private/public aspects, its adaptive/maladaptive applied characteristics, and present/past experiences. We examined these claims in a study using 602 young adults to whom the aforementioned scales were administered. Data were analyzed as follows: (1) correlation analysis to find simple associations between the measures; (2) factorial analysis using Oblimin rotation of total scores provided from the scales; and (3) factorial analysis considering the 102 items of the scales all together. It aimed to clarify relational patterns found in the correlations between SCSs, and to identify possible latent constructs behind these scales. Results support the adaptive/maladaptive aspects of self-consciousness, as well as distinguish to some extent public aspects from private ones. However, some scales that claimed to be theoretically derived from the concept of Private Self-Consciousness correlated with some of its public self-aspects. Overall, our findings suggest that while self-reflection measures tend to tap into past experiences and judged concepts that were already processed by the participants’ inner speech and thoughts, the Awareness measure derived from Mindfulness Scale seems to be related to a construct associated with present experiences in which one is aware of without any further judgment or logical/rational symbolization. This sub-scale seems to emphasize the role that present experiences have in self-consciousness, and it is argued that such a concept refers to what has been studied by phenomenology and psychology over more than 100 years: the concept of pre-reflective self-conscious.

## Introduction

Throughout the 20th century, Western Psychology has understood self-consciousness as an adaptive personality process that entails the natural human disposition of becoming an object of ones’ own consciousness (Duval and Wicklund, 1972; Wiley, 1994). Based on such definition, a number of scales related to self-consciousness have been produced in the recent years (see, for example, Trapnell and Campbell, 1999; Grant et al., 2002; Cardaciotto et al., 2008; McKenzie and Hoyle, 2008). As a consequence, it is assumed that there is a growing interest in empirical investigations on this construct. However, there is current discussion among cognitive scientists and philosophers on what constitutes self-consciousness (Hurley, 1997; Bermúdez, 1998; Gallup et al., 2002; Légrand, 2007; Gallagher and Zahavi, 2008; Metzinger, 2008; Zahavi, 2010).

Traditionally, the conceptualization behind self-consciousness measures relies on William James’ and George Mead’s definitions of self-consciousness. To become the object of one’s own attention, as suggested by Duval and Wicklund (1972), redirects us to the classical study of James (1890), who proposed that to reflect or think about the self requires that the subject (I) becomes the object (Me) of its own thoughts. From a social approach, Mead (1934) suggests that self-consciousness is the act of adopting the perspective of someone else (You) toward one’s own self (I). Currently, studies have argued about the distinction of self-consciousness between the act of reflection and the object of reflection (Düsing, 1997; Zahavi, 2010). Although psychological instruments that claim to measure facets of self-consciousness do not typically address such discussions, this study seeks to investigate which facets of self-consciousness are addressed by self-report measures and if there is consistency among the constructs defined by some scales and questionnaires that have currently been used in psychological studies.

In order to analyze the constructs that are usually addressed by self-consciousness measures, we will organize them into three sections, which correspond to three prominent facets of self-consciousness measured by some instruments: its private/public aspects, adaptive/maladaptive applied characteristics, and the present/past experiences in focus.

### Private/Public Aspects of Self-Consciousness

The public and private aspects of self-consciousness have been traditionally investigated and measured since the 1970s, when Fenigstein et al. (1975) developed the SCS. The theory behind it was proposed by Mead (1934) and was further operationalized as the theory of objective self-awareness by Duval and Wicklund (1972). The private and public self-consciousness constructs are distinguished based on the direction of the focus of one’s own attention, i.e., either inward (the inner feelings and beliefs one has toward oneself), or outward (the beliefs one has about what other people might think about them). This distinction has been criticized (Wicklund and Gollwitzer, 1987), yet many subsequent researchers have supported the differences between focusing on private and public self-characteristics (Franzoi et al., 1990; Grant, 2001; Eichstaedt and Silvia, 2003). Even so, other studies (Trapnell and Campbell, 1999; Grant et al., 2002) have used the SCS measures and focused specifically on its private aspect. In contrast, McKenzie and Hoyle (2008) discussed the presence of negative public and private aspects of self-consciousness as sustained and inflexible self-focused attention, which is also known as self-absorption.

### Adaptive/Maladaptive Aspects of Self-Consciousness

The adaptive and maladaptive aspects of self-consciousness emerged as a research topic mainly in the 1990s, reflecting concerns that the attention toward one’s self could be associated with both psychological mindedness and well-being (Trudeau and Reich, 1995), as well as with psychological distress (Ingram, 1990; Thomsen et al., 2013) and negative mood states (Wood et al., 1990). The fact that high levels of self-consciousness could be either associated with psychological well-being as well as psychological distress is usually described in the literature as the paradox of self-consciousness (Trapnell and Campbell, 1999; McKenzie and Hoyle, 2008; Simsek, 2013). In this sense, researchers claimed it became necessary to distinguish between the profits related to being aware of one’s thoughts and beliefs (the adaptive side of being self-aware), and counterproductive aspects of self-focus not being able to advance the critical thinking (its maladaptive facet).

According to Ingram (1990), psychological distress occurres when ones self-attention was inflexible, thus self-absorption was the product of disproportional, inappropriate, and excessive focus on self-attention. McKenzie and Hoyle (2008) created the Self-Absorption Scale (SAS), which measures the private and public facets of self-absorption. To other researchers, maladaptive self-attention occurs in the context of self-regulation processes, when discrepancies between one’s self-evaluative contents and their standards produce a negative affect and, as a consequence, negative psychopathological states (Buss, 1980; Fleckhammer, 2009). Thus, negative affect which is known to be associated with depression and anxiety generates a neurotic self-attentiveness. This neurotic self-attention was called self-rumination by Trapnell and Campbell (1999), and constituted the basis of their instrument, the Reflection–Rumination Questionnaire (RRQ). Rumination was defined as thoughts that frequently recur and are usually unwelcome. In contrast, self-absorption was, by definition, not only related to thoughts, but to any state in which the focus of all of one’s internal processes (affect, cognition, attitudes, motives) is directed to the self excessively and in a sustained manner. Rumination was related to psychopathological traits such as neuroticism, whereas self-absorption appeared to be a more generic characteristic. According to Ingram (1990), it is “difficult to find a psychological disorder that is not characterized by a heightened degree of self-focused attention” (Ingram, 1990, p. 165). Yet, rumination has also been associated with artistic creativity, mainly within musicians (Jones et al., 2014).

### Past/Present Aspects of Self-Consciousness

The past/present aspects of self-consciousness refer to the temporal instance that qualifies the self-conscious experience. On one hand, self-consciousness is viewed as a reflexive experience, and, thus, as a synonym for self-reflection. To some researchers (Anderson et al., 1996; Creed and Funder, 1998; Silvia, 1999) self-reflection is considered a dimension of private self-consciousness. In this context, it is related to the activity of inspecting and evaluating one’s own thoughts, feelings and behaviors. In fact, the etymology of the word reflection (from Latin, reflexus) indicates to bend back, suggesting that the thought needs to have something to which it is related, like a glance through a past experience, so that it can exist. This is consistent with James’ (1890) idea of thought and the aforementioned distinction between “I” and “Me”. As such, to reflect or think about the self requires the subject to become the object of their own thoughts; thus, a reflection must refer to some content, an object that is located in past experience.

On the other hand, self-consciousness can be associated to a present moment of self-experience in which one is aware of their experience without any reflexive judgment attached, which is usually investigated in mindfulness studies. Theory and research on self-consciousness focused on the Eastern traditions, more specifically on the concept of mindfulness, have been having a growing interest in the psychological literature (Kabat-Zinn, 2003; Bishop et al., 2004; Rosch, 2007; Hanley and Garland, 2014). Mindfulness is considered to be different from other conscious states, such as self-concept, schemes, and other constructs related to self-reflection: it is solely related to the quality of the conscious experience in the very moment of its occurrence (Cardaciotto et al., 2008); therefore, it should not be associated with reflexive content (Shear and Jevning, 1999). Bishop et al. (2004) suggest that mindfulness has two main components: a sustained attention to the present moment (Awareness) and an open and acceptant attitude toward the experience (Acceptance). This way, consciousness in the present moment is understood as a continuous process of monitoring internal and external events.

Hence, two approaches to self-consciousness regarding its focus are here distinguished: one is related to a reflective instance and presupposes the content to which self-focus is addressed (which is hereby called the ‘past’ approach), while the other entails a non-themed consciousness of one’s present experience as a whole (which is called the ‘present’ approach). These two features of self-consciousness suggest two distinguishable epistemological focuses: one is focused on the content and information carried out by thoughts and memory (procedural cognition), and the other is focused on the phenomenal, embodied, and situated cognition.

### Research Aims

This present study aims to examine which of the facets (private/public aspects, adaptive/maladaptive applied characteristics, and the present/past experiences of self-consciousness) are being considered in self-consciousness self-report measures. Thus, it was expected that the three prominent facets of self-consciousness (private/public aspects, adaptive/maladaptive applied characteristics, and present/past experiences) would be distinguished by a factor analysis. Moreover, in previous validation and adaptation studies (DaSilveira et al., 2011, 2012a,b) in which five SCSs were applied altogether, the authors received feedback from participants claiming that some items from different instruments appear to be very similar and repetitive; thus, we sought to investigate which items from different scales might reflect the same facets underlying self-consciousness. Scales that measure self-consciousness and its related constructs were chosen for their traditional and frequent use in psychological research or for their recent innovation. Five instruments were selected: (1) the SCS (Revised; Scheier and Carver, 1985); (2) the RRQ (Trapnell and Campbell, 1999); (3) the SRIS – Self-Reflection and Insight Scale (Grant et al., 2002); (4) the SAS (McKenzie and Hoyle, 2008); and (5) the PHILMS – Philadelphia Mindfulness Scale (Cardaciotto et al., 2008). The scales are described in the following section.

Considering the complex relationships found among constructs measured by these scales, our expectations for this research are the following:

(1) A positive association between the constructs of Private Self-Consciousness (SCS) with Reflection (RRQ), Self-Reflection (SRIS), and Private Self-Absorption;
(2) A moderate positive association between Private Self-Consciousness with Rumination (RRQ); and a negative association with Insight (SRIS);
(3) No associations among the constructs Rumination and Reflection (RRQ), and Self-Reflection and Insight (SRIS) with the public aspects of both Self-consciousness and Self-Absorption;
(4) Positive associations between Public Self-consciousness (SCS) with Social Anxiety, and the public aspect of Self-Absorption;
(5) Positive correlation between the Private Self-Absorption (from SAS) and Rumination (from RRQ), as both claim to measure negative aspects of private self-consciousness;
(6) Inverse association between Rumination (from RRQ) and Insight (from SRIS), considering that the authors (Roberts and Stark, 2008) claim that counter-intuitively high levels of self-reflection may be an impediment to developing insight.
(8) Moderate positive association between the construct Reflection (RRQ) with the constructs Acceptance and Awareness (PHILMS), since reflection disclose an inquisitive activity in self-consciousness.

## Materials and Methods

### Sample and Procedures

The participants were recruited from the general population in Brazil using a snowball sampling method. An initial sample was asked to recruit people from their environment who would be willing to take part in this study. Six hundred and two individuals were recruited, with a mean age of 25.4 years (SD = 9.63). Although not all participants were in direct contact with the researcher and we lack control of the sampling method, a limitation of snowball form of recruitment, it allowed us to have a cost-efficient number of participants from all Brazilian regions: South 69%, Southeast 20%, Northeast 5%, Central-West 1.2%, North 4%. These Brazilian macroregions are composed of states with similar cultural, economical, historical and social aspects, and they are the most widely referred in Brazil because official information given by the Brazilian Institute for Geography and Statistics uses this system. In addition, 4% of respondents were residing abroad at the time of the survey. Of the participants 36.4% were graduate students, 28.2% undergraduate students, 25, 6% had a university degree, and 5% had completed high school. The rest of the participants (4.8%) did not report their schooling. Of the cohort, 63% were women and 37% men. Questionnaires were answered anonymously, and participants filled out a consent form before taking part in the study. The study was a within-subjects correlational design, and it was approved by the ethics committee of a Brazilian public University, where this study was conducted.

### Measurements

#### The Self-Consciousness Scale – Revised (SCS-R)

The SCS-R (Scheier and Carver, 1985) is a revised version of the SCS (Fenigstein et al., 1975). The theory behind it was proposed by Mead (1934) and further operationalized as the theory of objective self-awareness by Duval and Wicklund (1972). It understands self-consciousness as the activity of becoming the object of one’s own thoughts, and claims to measure three constructs related to self-consciousness. Private Self-Consciousness is related to the inward direction of one’s thoughts, whereas Public Self-Consciousness is related to the outward direction of one’s thoughts, or the ideas and beliefs one has about the impact of their presence on other people. The third subscale is called Social Anxiety and is considered an enfoldment of the Public Self-Consciousness subscale. The authors defined this construct as a consequence that could emerge from some further reflection upon one’s own public self-consciousness. Historically, the SCS was extended and later revised by Buss (1980), Carver and Scheier (1981), Pyszczynsky and Greenberg (1987), and Grant (2001). This Scale continues to be adapted for different populations, like the example of its recent version for use with children (Takishima-Lacasa et al., 2014). It originally consists of 23 items measured on a five-point Likert scale, which were divided into three dimensions: Private Self-Consciousness (nine items, such as “I’m always trying to figure myself out”), Public Self-Consciousness (seven items, such as “I’m concerned about the way I present myself”), and Social Anxiety (six items, such as “I have trouble working when someone is watching me”). The Brazilian version used in this study has 22 items and was adapted by Teixeira and Gomes (1995). It presented a sufficient reliability score (α = 0.73 and.89 for test–retest), and confirmed the tri-factor structure in accord with the original scale.

### The Reflection–Rumination Questionnaire (RRQ)

The RRQ (Trapnell and Campbell, 1999) was built based on the SCS and aims to measure two forms of the Private Self-Consciousness subscale: Self-reflection and Self-rumination, to which the authors also refer to as “Reflection” and “Rumination.” In this scale, Reflection was defined in contrast to self-rumination, as a form of self-attention that infers curiosity and entails a philosophical interest in the self, whereas Rumination is the counterproductive aspect of self-reflection. The RRQ has 24 items that are equally divided into Reflection (for example, “My attitudes and feelings about things fascinate me”) and Rumination (for example, “My attention is often focused on aspects of myself I wish I’d stop thinking about”). The Brazilian version used in this study was adapted by Zanon and Teixeira (2006) and presented a good reliability score (α = 0.87), in addition to confirming the two-factor structure in accord with the original scale.

### The Self-Absorption Scale (SAS)

According to McKenzie and Hoyle (2008), the concept of self-absorption is defined as a sustained and inflexible self-focused attention, which constitutes a psychopathological aspect of self-consciousness. To the authors of the SAS, their concept differs from what is measured by the RRQ, once the Trapnell and Campbell’s (1999) measure is said to be an exclusive bifurcation from the Private Self-Consciousness subscale by Scheier and Carver (1985). The special attention given to the distinction between public and private aspects of self-consciousness in the SAS is justified by the authors since such distinction has been useful to detect self-conscious emotions and it could contribute in order to integrate the self-absorption construct to the literature on self-consciousness and psychopathology. The original English version of the SAS consists of 17 items, divided into two dimensions. One dimension is related to the private instance of self-absorption and consists of eight items, such as “I think about myself more than anything else.” The other dimension is related to the public instance of self-absorption, and has nine items, such as “I find myself wondering what others think of me even when I don’t want to.” The Brazilian version of the SAS used in this study was adapted by DaSilveira et al. (2011) and had a good reliability score (α = 0.83), with one item excluded from the Private Self-Absorption subscale. Thus, the Brazilian version of this scale has 16 items (seven for Private Self-Absorption and nine for Public Self-Absorption).

### The Self-Reflection and Insight Scale (SRIS)

The SRIS (Grant et al., 2002) was originally constructed based on the Private Self-Consciousness dimension from the SCS (Scheier and Carver, 1985), and its authors claim that it is an updated version of its Private Self-Consciousness subscale. The authors added the Insight dimension based on findings that had shown another dimension related to Private Self-Consciousness, called the internal state of awareness (Anderson et al., 1996; Creed and Funder, 1998; Silvia, 1999). This dimension was related to a state of internal understanding that one has toward one’s own thoughts, feelings, and behaviors. This definition was also used by Grant et al. (2002) to describe the Insight scale. The SRIS has 12 items for Self-reflection (for example, “I frequently take time to reflect on my thoughts”) and eight items for Insight (for example, “I am often aware that I am having a feeling, but I often don’t quite know what it is,” which is a reversed item). The Brazilian version used in this study was adapted by DaSilveira et al. (2012a) and presented satisfactory reliability, with an α = 0.90 for Self-reflection and 0.82 for Insight, respectively, 0.91 and 0.87 in the original scale. The 2-factor structure of the scale was confirmed.

### The Philadelphia Mindfulness Scale (PHILMS)

The PHILMS (Cardaciotto et al., 2008) was developed to measure mindfulness as a psychological construct. Concepts as mindfulness and self-consciousness are target of a vast discussion in the literature (Bishop et al., 2004). Its meaning is related to the clarity and non-evaluative fluctuation of the attention toward experience (Kabat-Zinn, 2003), with roots in both Buddhist Psychology and meditation practices. In psychological literature, mindfulness is usually distinguished from conscious states such as self-concept, schemas, and narratives, which have a reflexive judgment attached to it (Shear and Jevning, 1999). Cognitive scientists have struggled to define mindfulness in a way it can be operationally viable for measurement and training (Van Dam et al., 2010). In this tradition, PHILMS stands out as a scale that operationalised mindfulness as a psychological process, targeting populations unfamiliar with meditation practices. PHILMS accounts for the two main components of mindfulness suggested by Bishop et al. (2004): sustaining attention to the present moment (Awareness), and openness and non-attachment to the experience (Acceptance). More specifically, Awareness is related to a continuous monitoring of internal and external events of experience, in a wider sense than just focal attention. Acceptance is believed to put the PHILMS back to the Buddhist tradition concepts of acceptance and detachment, since it describes the experience of events without judgements and interpretation. But, in order to present the construct in an accessible way to participants that are not familiar to Buddhist practices, the authors reversed the items meant to measure it. The result is a group of 10 items formulated in sentences as “I try to distract myself when I feel unpleasant emotions.” The complete PHILMS consists of 20 items measured by a five-point Likert scale that were originally divided into two dimensions: Acceptance (10 items), and Awareness (10 items, such as “When I walk outside, I am aware of smells or how the air feels against my face”). The Brazilian version of the scale used in this study was adapted by DaSilveira et al. (2012b) and presented satisfactory reliability, with confirmed factor structure (α = 0.81 for Awareness, and 0.85 for Acceptance, same as the original scale).

### Data Analysis

Correlations between scores on the subscales of the five instruments were calculated. The reliability of the measures was calculated using Cronbach’s alpha. In order to reveal latent structures behind the five SCSs, a Factorial Analysis with Oblimin rotation was calculated, using both total scores provided by the scales and all scale items. All analyses were conducted using the statistical package program SPSS version 11.

## Results

**Table 1** presents means, SDs, and internal reliabilities for the measures used in the study. The reliabilities were adequate to good, but some measures clearly had greater internal reliability than others (e.g., Self-reflection vs. Public and Private Self-Consciousness). Although not a main hypothesis, independent sample *t*-tests examined whether there were any sex differences across the measures. The results showed that women had higher scores than men for self-reflection [SRIS; *t*(155) = -2.86; *p* < 0.005].

**Table 1:** Descriptive statistics and subscale reliabilities.

Measure	Mean	SD	Cronbach’s α
Private Self-Consciousness [Self-Consciousness Scale - Revised (SCS-R)]	34.18	5.06	0.74
Public Self-Consciousness (SCS-R)	27.22	3.91	0.74
Social Anxiety (SCS-R)	17.96	5.67	0.84
Reflexion [Reflection-Rumination Questionnaire (RRQ)]	45.34	9.49	0.89
Rumination (RRQ)	41.36	9.47	0.88
Self-Reflection [Self-Reflection and Insight Scale (SRIS)]	48.79	8.53	0.90
Insight (SRIS)	26.43	6.08	0.81
Private Self-Absorption [Self-Absorption Scale (SAS)]	13.12	5.20	0.82
Public Self-Absorption (SAS)	25.37	6.93	0.83
Acceptance [Philadelphia Mindfulness Scale (PHILMS)]	29.18	7.10	0.86
Awareness (PHILMS)	36.57	6.11	0.82

**Table 2** presents correlations between all study measures. Although the negative correlations between Private Self-Consciousness and Acceptance, Public Self-Consciousness and Insight, Public Self-Consciousness and Acceptance, Social Anxiety and Insight, Social Anxiety and Acceptance, and Private Self-Absorption and Acceptance were statistically significant, they were low in magnitude. Because of the sample size, we hereby highlight correlations of moderate magnitudes (i.e., *r* > 0.40, according to the Dancey and Reidy, 2011, suggested parameters) in **Table 2**.

**Table 2:** Correlations for the self-consciousness related subscales.

	PRSC	PBSC	SA	REFL	RUM	SR	INS	PRSA	PBSA	ACC	AWA
PRSC	-	**0.40^∗^**	0.13^∗∗^	**0.64^∗^**	0.34^∗^	**0.68^∗^**	-0.09	0.33^∗^	0.19^∗^	-0.13^∗∗^	0.37^∗^
PBSC		-	0.19^∗^	0.16^∗^	0.39^∗^	0.28^∗^	-0.14^∗∗^	0.16^∗^	**0.40^∗^**	-0.19^∗^	0.21^∗^
SA			-	-0.01	**0.42^∗^**	-0.01	-0.33^∗^	0.24^∗^	**0.44^∗^**	-0.29^∗^	-0.07
REFL				-	0.11	**0.68^∗^**	0.03	0.22^∗^	-0.02	0.09	0.27^∗^
RUM					-	0.22^∗^	-**0.52^∗^**	**0.47^∗^**	**0.60^∗^**	-**0.47^∗^**	-0.02
SR						-	0.06	0.22^∗^	0.05	-0.05	0.36^∗^
INS							-	-**0.42^∗^**	-**0.49^∗^**	**0.51^∗^**	0.22^∗^
PRSA								-	**0.49^∗^**	-0.31^∗^	0.01
PBSA									-	-**0.42^∗^**	-0.03
ACC										-	-0.05
AWA											-

*^∗^Correlation is significant at the 0.01 level (two-tailed).*

*^∗∗^Correlation is significant at the 0.05 level (two-tailed).*

*Acronyms legend: PRSC, Private Self-Consciousness (SCS-R); PBSC, Public Self-Consciousness (SCS-R); SA, Social Anxiety (SCS-R); REFL, Reflection (RRQ); RUM, Rumination (RRQ); SR, Self-Reflection (SRIS); INS, Insight (SRIS); PRSA, Private Self-Absorption (SAS); PBSA, Public Self-Absorption (SAS); ACC, Acceptance (PHILMS); AWA, Awareness (PHILMS).*

*All values in bold are correlations higher than 0.40.*

As expected, there were moderate positive associations between Private Self-Consciousness, Reflection and Self-Reflection. And there were low positive associations between Private Self-Consciousness, Rumination and Private Self-Absorption. Low positive associations were also found between Private Self-Consciousness and the two public dimensions of both Self-Consciousness and Self-Absorption, contrary to theoretical expectations. Social Anxiety also had low associations with both Private and Public Self-Consciousness, although it was only expected to correlate with the later.

In accord with the hypotheses, Rumination had a modest negative association with Insight. A low correlation was also found between Rumination and Private Self-Absorption. Surprisingly, there was a high association between Rumination and the public dimension of self-absorption. In fact, Rumination was correlated with the public dimensions subscales (Public Self-Consciousness and Public Self-Absorption) than the private dimensions subscales (Private Self-Consciousness and Private Self-Absorption).

For the last part of the correlational hypothesis, there were low and negative significant correlations between Rumination and Acceptance. However, the Awareness correlation with Rumination was negative but not significant. In fact, the Awareness subscale had low significant correlations with Private Self-Consciousness, Public Self-Consciousness, Reflection, Insight, and Self-Reflection.

As observed in the correlational analyses, several expected theoretical and empirical hypotheses based on previous studies were maintained. However, several theoretical hypotheses were contradicted, as in the public/private aspects of self-consciousness. Private Self-Consciousness, Public Self-Consciousness, Private Self-Absorption, and Public Self-Absorption did not react as expected in most analyses. On the other hand, the maladaptive/adaptive distinction involving the Rumination, Private Self-Absorption, and Public Self-Absorption scales was maintained without contradiction.

To clarify the relationship patterns found in the correlations between the self-consciousness measures and identify latent constructs, total scores from the 11 subscales were subjected to a factor analysis, using Oblimin rotation. The Kaiser-Meyer-Olkin (KMO) measure verified the sampling adequacy for the analysis, KMO = 0.78. Bartlett’s Test of Sphericity χ^2^ (55) = 1528.78, *p* < 0.001. Three factors with an Eigenvalue greater than 1 were extracted. **Table 3** shows the loadings for each scale on the relevant factor and the variance explained by the factor. Together, these factors account for 63.3% of the variance. Interestingly, the first rotated factor displayed all measures related to the maladaptive dimensions of self-consciousness (Social Anxiety, Rumination, and Private and Public Self Absorption), plus the variables of the subscales Insight and Acceptance with negative scores. The second factor had high positive loadings for the adaptive characteristics of self-reflection [i.e., Private Self-Consciousness, Reflection (from RRQ) and Self-Reflection (SRIS)]. The third factor stands out public self-consciousness subscale following by the private self-consciousness subscale with lower scores. Interestingly, the subscale of public self-absorption was the third highest scoring in that factor (0.32) very close to the cut-off point of 0.35. The subscale Awareness did not significantly load on any factor. Public Self-Consciousness, which initially loaded on both Factors 2 and 3, appeared closer to F2 in the Factor Plot.

**Table 3:** Pattern loadings for the principal axis factoring analysis with Oblimin rotation and Kaiser Normalization.

Measure	F1	F2	F3
Private Self-Consciousness (SCS-R)		**0.82**	**0.45**
Public Self-Consciousness (SCS-R)			**0.61**
Social Anxiety (SCS-R)	**0.50**		
Reflection (RRQ)		**0.80**	
Rumination (RRQ)	**0.74**		
Self-Reflection (SRIS)		**0.60**	
Insight (SRIS)	-**0.56**		
Private Self-Absorption (SAS)	**0.62**	0.35	
Public Self-Absorption (SAS)	**0.78**		0.32
Acceptance (PHILMS)	-**0.55**		
Awareness (PHILMS)			0.30
Eigenvalue	3.4	2.1	1.0
Variance (%)	31.7	19.5	9.1
Total Variance = 60.4%			
Factor correlation matrix: F1	1.0	0.11	0.10
Factor correlation matrix: F2		1.0	0.27
Factor correlation matrix: F3			1.0

*All values in bold are correlations higher than 0.40.*

All 102 items from the 11 subscales were subjected to a factor analysis using Oblimin rotation. Twenty-two factors with Eigenvalues greater than 1 and 10 factors with Eigenvalues greater than 1.75 were extracted (O’Connor, 2000). Together, the 22 factors account for 63.4%, while 11 factors account for 50.3% of the variance. According to O’Connor (2000), we used a parallel analysis engine to aid in determining the number of factors to retain (suggested by Patil et al., 2007), and it suggested proceeding a factorial analysis retaining four factors. This procedure aimed to identify how the items would react when forced to fit the smaller number of factors.

The 4-factor solution had a KMO = 0.89, Bartlett’s Test of Sphericity χ^2^ (5151) = 23932.9, *p* < 0.001. The distribution of items of each Scale according to the factor in which they loaded can be seen on **Table 4**. Since this analysis includes 102 items, the authors chose to display only the factor loadings higher than 0.35. Some items loaded in more than one factor, as noted on **Table 4**. Factor 1 could be called “reflection”, since it collected the items related to the adaptive and healthy facet of reflecting upon one’s own self. It grouped together all the items from the Reflection and Self-Reflection subscales (from the RRQ and SRIS scales, respectively), as well as items from the Private SCS. Examples of items in Factor 1 are: “I am always trying to figure myself out” (Private Self-Consciousness subscale), all items from the Reflection subscale of the RRQ as “I love analyzing why I do things,” and all items from the Self-Reflection subscale of the SRIS, as “It is important for me to evaluate the things that I do.” Moreover, one single item of the private aspect of the SAS also belongs to this factor, the item “I think about myself more than anything else.”

**Table 4:** Suggested four-factor model (pattern loadings) for the 102 items of five Scales (Principal Axis Factoring with Oblimin Rotation and Kaiser Normalization).

Scales Factors	Private and Public Self-Consciousness and Social Anxiety (SCS)			Self-Reflection and Insight (SRIS)			Private and Public Self-Absorption (SAS)			Reflection–Rumination (QRR)			Acceptance and Awareness PHILMS		
	Item	F. L.	Ss	Item	F. L.	Ss	Item	F. L.	Ss	Item	F. L.	Ss	Item	F. L.	Ss
F1 – Reflection (Adaptive and health facet of self-consciousness) Eigenvalue 14.507 % of Variance 14.22 Factor correlation with F2: 0.16 F3: -0.13 F4: -0.38	1 4 8 12 14 17	(0.65) (0.67) (0.53) ∗(0.45) (0.53) (0.46)	PRSC PRSC PRSC PRSC PRSC PRSC	1 2 5 7 8 10 12 13 15 16 18 19	(0.54) (0.55) (0.58) (0.72) (0.71) (0.55) (0.70) (0.53) (0.67) (0.58) (0.65) (0.62)	SR SR SR SR SR SR SR SR SR SR SR SR	4	(0.37)	PRSA	13 14 15 16 17 18 19 20 21 22 23 24	(0.53) (0.57) (0.72) (0.50) (0.67) (0.68) (0.36) (0.58) (0.59) (0.56) (0.66) (0.69)	REFL REFL REFL REFL REFL REFL REFL REFL REFL REFL REFL REFL			
F2-Maladaptive facet of self-consciousness Eigenvalue 12.09 % of Variance 11.86 Factor correlation with F3: 0.20 F4: -0.002	2 3 5 6 7 9 13 15 16 18 22	(0.37) (0.51) (0.49) (0.39) (0.41) (0.50) (0.41) (0.40) (0.34) (0.64) (0.41)	PBSC SA PBSC PRSC SA SA PBSC SA PBSC PBSC SA				1 2 3 7 8 10 13 16 17	(0.62) (0.46) (0.53) (0.42) (0.44) (0.54) (0.43) (0.50) (0.58)	PBSA PBSA PBSA PBSA PBSA PBSA PBSA PBSA PBSA	1 2 3 4 5 6 7 8 9 10 11 12	(0.44) ∗(0.69) ∗(0.54) (0.57) ∗(0.65) (0.46) (0.60) (0.59) (0.49) ∗(0.39) (0.51) ∗(0.61)	RUM RUM RUM RUM RUM RUM RUM RUM RUM RUM RUM RUM			
F3 – Avoidance Eigenvalue 3.93 % of Variance 3.85 Factor correlation with F4: 0.10				4 9 11 14 17 20	(0.57) (0.36) (0.45) (-0.56) (-0.61) ∗(0.36)	INS INS INS INS INS INS	5 6 9 11 15	(-0.37) (-0.41) (-0.39) (-0.47) (-0.49)	PRSA PRSA PRSA PRSA PRSA	2 3	∗(-0.49) ∗(-0.44)	RUM RUM	2 4 6 8 10 12 14 16 18 20	(0.30) (0.57) (0.65) (0.58) (0.66) (0.68) (0.49) (0.63) (0.40) (0.53)	ACC ACC ACC ACC ACC ACC ACC ACC ACC ACC
F4- awareness of self/ awareness of experience”. (being fully wake or present) Eigenvalue 2.85 % of Variance 2.80	10 12 19 20 21	(0.42) ∗(0.44) (0.45) (0.44) (0.45)	PBSC PRSC PRSC PBSC PRSC	3 6 20	(0.56) (0.47) ∗(0.51)	INS INS INS				5 10 12	∗(0.36) ∗(0.38) ∗(0.38)	RUM RUM RUM	1 3 5 7 9 11 13 15 17 19	(0.54) (0.40) (0.35) (0.40) (0.41) (0.57) (0.53) (0.41) (0.64) (0.58)	AWA AWA AWA AWA AWA AWA AWA AWA AWA AWA

*Legend:*

*Item: Number of the item on its correspondent Scale.*

*F.L.: Factor Loadings – values higher than 0.35 are presented between parentheses.*

*∗Indicates items that appear in more than one factor.*

*Ss: Sub-scale - acronyms legend for each sub-scale: PRSC, Private Self-Consciousness (SCS-R); PBSC, Public Self-Consciousness (SCS-R); SA, Social Anxiety (SCS-R); REFL, Reflection (RRQ); RUM, Rumination (RRQ); SR, Self-Reflection (SRIS); INS, Insight (SRIS); PRSA, Private Self-Absorption (SAS); PBSA, Public Self-Absorption (SAS); ACC, Acceptance (PHILMS); AWA, Awareness (PHILMS).*

Factor 2 could be called the maladaptive facet of self-consciousness, since it grouped items that refer to ruminative thoughts and some sense of avoidance toward thoughts and the act of driving one’s attention toward themselves, either in private or public situations. It includes items such as “I’m concerned about my style of doing things” (from the Private Self-Consciousness subscale), “I feel anxious when I speak in front of a group” (from the Social Anxiety subscale), “I’m concerned about the way I present myself” (from the Public Self-Consciousness subscale), “I find myself wondering what others think about me even when I don’t want to” and “When I start thinking about how others see me, I get all worked up” (both from the Public Self-Absorption subscale), “I tend to ‘ruminate’ or dwell over things that happen to me for a really long time afterward.” and “I often reflect on episodes of my life that I should no longer concern myself with” (both from the RRQ’s Rumination subscale).

Factor 3 could be called avoidance, since it gathered all items related to insights and impediments in deliberations one deals toward their own thoughts and behaviors. This factor groups together all reversed items from the Insight subscale, such as “My behavior often puzzles me” and “I often find it difficult to make sense of the way I feel about things,” as well as all Private Self-Absorption items, such as “When I have to perform a task, I do not do it as well as I should because my concentration is interrupted with thoughts of myself instead of the task,” “My mind never focus on things other than myself for very long.” It also has all the items from the Acceptance subscale, such as “When I have a bad memory, I try to distract myself to make it go away” and “I tell myself that I shouldn’t feel sad.”

Finally, Factor 4 could be called “awareness of self/ awareness of experience,” since it not only gathered all the items from the Awareness subscale (such as “I am aware of what thoughts are passing through my mind” and “When I walk outside, I am aware of smells or how the air feels against my face”) but it also collects all the items that either describe this present moment of experiences one is having or explicitly contain the verb “aware” in their descriptions, such as “I usually know why I feel the way I do” and “I am usually aware of my thoughts” (both from the SRIS), and “I’m alert to changes in my mood” and “I’m usually aware of my appearance” (both items from the SCS).

## Discussion

As for the correlational analyses, Insight scores were negatively correlated with Rumination, which corroborates the theoretical assumption proposed by Roberts and Stark (2008). Similarly, both Acceptance and Insight scores had a modest positive correlation (*r* = 0.51) and negatively loaded on the same factor in our first Factorial Analysis. In addition, Factor 2 was composed of items that are related to self-consciousness as a self-reflective activity, including the scores of Reflection (RRQ) and Self-Reflection (SRIS) next to the Private Self-Consciousness subscale. Since the Awareness scores did not load on the same factor, we suggest that the Awareness component of mindfulness, as measured by the PHILMS, may not reflect self-reflection. Indeed, the Awareness subscale scores had also presented significant but low correlations with self-reflective measures. The fact that the correlation between Factor 1 and Factor 4 was -0.38 could be used to support the argument that self-reflection is distinct from Awareness. Thus, Awareness appeared to resemble a construct related to the present experience of which one is cognizant without any further judgment nor logical/rational symbolisation.

The Factorial Analysis from the 11 subscale scores provided interesting empirical evidence supporting the maladaptive/adaptive distinction in self-consciousness measures. Factors 1 and 2 have gathered either the counterproductive aspects of self-consciousness or its aspect associated with psychological mindedness and well-being. Note that scores of Public SCS loaded on a separate factor alongside with Private Self-Consciousness. Scores for the Public aspects of Self-Absorption loaded on the same factor but did not reach the minimal loading (i.e., 0.35).

A very similar pattern could be observed in the Factorial Analysis of the 102 items altogether. Items from Factors 1 and 2 reflected the adaptive/maladaptive aspects of self-consciousness. Self-consciousness private aspects predominantly carried out in Factor 1, while the public aspects concentrated in Factor 2. Factor 3 brought a consistent association of one’s promptitude for discernment of problems and situations (Insight), combined with one’s openness to inner and outer experiences (Acceptance), as well as a sustained and inflexible inward self-focused attention (Private Self-Absorption). It is interesting to note that the Insight items as well as the items from Private Self-Absorption were negative; i.e., they were together in the same factor, but still on the opposite quadrant when compared to the Acceptance items. Such behavior makes theoretical sense, hence Insight and Private Self-Absorption are both constructs related to judging the content of one’s thoughts, whereas Acceptance is related to non-judgemental openness to experiences, but, it is described in the PHILMS scale as reversed items; thus, it is also referring to acts that people entail in order to block such judgmental thoughts toward their experiences. Factor 4 offers some evidence for open-mindedness, the receptiveness to the present experience and to new ideas. Thus, in the empirical analyses of the scales, two out of the three prominent facets of self-consciousness were prominent: the adaptive/maladaptive applied characteristics, and the present/past experiences that are focused by the self.

### Final Considerations

Self-consciousness, as a construct that is assessed by self-report measures, can be distinguished by its private and public aspects, adaptive/maladaptive applied characteristics, and the present/past experiences that are focused by the self. In this study, we compared these three components by examining their associations with each other (**Table 3**). The findings suggested that it is possible to argument in favor of a distinction between the public and private aspect of self-consciousness. The projection of public self-consciousness (**Table 3**, third factor) is notorious because most of these scales are explorations of the Private Self-Consciousness. The distinction between adaptive and maladaptive dimensions of self-reflection was sustained by the correlation as well as the factor analyses. Nevertheless, the data presented should also be tested with a neuroticism measure to identify the underlying factor behind such differences. Additionally, in all analyses (correlations and factor analysis), it was observed that the Awareness subscale of the PHILMS was always distinguishable, which suggests that it can be an evidence of pre-reflective aspects of self-consciousness. Moreover, it demonstrates the capacity of human consciousness to establish the condition of here and now, which is the sense of present moment.

When analyzing the factor structure for the total subscale scores, Insight behaved differently than other self-reflective constructs. In the factor analysis of all items, many items from the Insight subscale negatively loaded on the same factor as the maladaptive items. As previously mentioned, such findings corroborated several theoretical expectations (Roberts and Stark, 2008). However, Grant et al. (2002) stated that both subscales, Self-Reflection and Insight, were sub-dimensions of the private self-consciousness construct. Thus, it should be expected that Insight would load in the same factor as Self-Reflection. According to Grant et al. (2002) Insight was a synonym for an internal state of awareness (Anderson et al., 1996; Creed and Funder, 1998; Silvia, 1999). This state has clear theoretical similarities to what the Awareness subscale claims to measure, which suggests a need for further studies to clarify the differences between the Awareness and Insight, and determine which measure accounts for the internal state of awareness, as noted (Creed and Funder, 1998).

In summary, our findings suggest: (1) that in a non-clinical sample, both adaptive/maladaptive and past/present dimensions of self-consciousness were relatively stable structures; and (2) that in spite of variations in the formulation of the scales items, the structural model of Self-Consciousness in James and Mead prevails the subject (I) becomes the object (Me) of its own thoughts. Further studies are needed to confirm that the awareness factor measures what the phenomenology tradition understands as pre-reflective self-consciousness.

## Conflict of Interest Statement

The authors declare that the research was conducted in the absence of any commercial or financial relationships that could be construed as a potential conflict of interest.
